# Supplementary material for: Changes in background characteristics and risk factors among SIDS infants in England: cohort comparisons from 1993 to 2020
Source: BMJ Open. 2023 Oct 13;13(10):e076751. doi: 10.1136/bmjopen-2023-076751 (PMC10582842; doi:10.1136/bmjopen-2023-076751)
Supplement: Author's manuscript [file bmjopen-2023-076751.draft_revisions.pdf]

BMJ Open

BMJ Open is committed to open peer review. As part of this commitment we make the peer review history of every article we publish publicly available.

When an article is published we post the peer reviewers' comments and the authors' responses online. We also post the versions of the paper that were used during peer review. These are the versions that the peer review comments apply to.

The versions of the paper that follow are the versions that were submitted during the peer review process. They are not the versions of record or the final published versions. They should not be cited or distributed as the published version of this manuscript.

BMJ Open is an open access journal and the full, final, typeset and author-corrected version of record of the manuscript is available on our site with no access controls, subscription charges or pay-per-view fees (http://bmjopen.bmj.com).

If you have any questions on BMJ Open's open peer review process please email info.bmjopen@bmj.com

Changes in background characteristics and the prevalence of SIDS risk factors in England

Journal:	*BMJ Open*
Manuscript ID	bmjopen-2023-076751
Article Type:	Original research
Date Submitted by the Author:	16-Jun-2023
Complete List of Authors:	Pease, Anna; University of Bristol Medical School, Population Health Sciences<br>Turner, Nicholas; University of Bristol Medical School, Population Health Sciences<br>Ingram, Jenny; University of Bristol Medical School, Population Health Sciences<br>Fleming, Peter; University of Bristol Medical School, Population Health Sciences<br>Patrick, Karen; Royal United Hospital Bath NHS Trust<br>Williams, Tom; University of Bristol Medical School, Translational Health Sciences<br>Sleap, Vicky; University of Bristol Medical School, Translational Health Sciences<br>Pitts, Kieren; University of Bristol, Research IT<br>Luyt, Karen; University of Bristol Medical School, Translational Health Sciences<br>Ali, Becky; University of Bristol Medical School, Population Health Sciences<br>Blair, Peter; University of Bristol Medical School, Population Health Sciences
Keywords:	Cot death < PAEDIATRICS, EPIDEMIOLOGY, Community child health < PAEDIATRICS, PUBLIC HEALTH

I, the Submitting Author has the right to grant and does grant on behalf of all authors of the Work (as defined in the below author licence), an exclusive licence and/or a non-exclusive licence for contributions from authors who are: i) UK Crown employees; ii) where BMJ has agreed a CC-BY licence shall apply, and/or iii) in accordance with the terms applicable for US Federal Government officers or employees acting as part of their official duties; on a worldwide, perpetual, irrevocable, royalty-free basis to BMJ Publishing Group Ltd ("BMJ") its licensees and where the relevant Journal is co-owned by BMJ to the co-owners of the Journal, to publish the Work in this journal and any other BMJ products and to exploit all rights, as set out in our licence.

The Submitting Author accepts and understands that any supply made under these terms is made by BMJ to the Submitting Author unless you are acting as an employee on behalf of your employer or a postgraduate student of an affiliated institution which is paying any applicable article publishing charge ("APC") for Open Access articles. Where the Submitting Author wishes to make the Work available on an Open Access basis (and intends to pay the relevant APC), the terms of reuse of such Open Access shall be governed by a Creative Commons licence – details of these licences and which Creative Commons licence will apply to this Work are set out in our licence referred to above.

Other than as permitted in any relevant BMJ Author's Self Archiving Policies, I confirm this Work has not been accepted for publication elsewhere, is not being considered for publication elsewhere and does not duplicate material already published. I confirm all authors consent to publication of this Work and authorise the granting of this licence.

For peer review only

Changes in background characteristics and the prevalence of SIDS risk factors in England

Anna S Pease[1], Nicholas Turner[1], Jenny Ingram[1], Peter J Fleming[1], Karen Patrick[2], Tom Williams[3], Vicky Sleap[3], Kieren Pitts[4], Karen Luyt[3], Becky Ali[1], Peter S Blair[1]

Corresponding author: Dr Anna S Pease, Population Health Sciences, Bristol Medical School, University of Bristol, UK. Email: a.pease@bristol.ac.uk

[1] Population Health Sciences, Bristol Medical School, University of Bristol, UK.

[2] Royal United Hospitals, Bath, UK.

[3] Translational Health Sciences, Bristol Medical School, University of Bristol, Bristol, UK

[4] Research IT, University of Bristol, UK

For peer review only

Abstract

Objectives: Using the National Child Mortality Database (NCMD), this work aims to investigate background characteristics and risk factors in the sleeping environment associated with SIDS (sudden infant death syndrome) and compare the prevalence with previous English SIDS case-control studies.

Design: Cohort of SIDS in 2020 compared to a combined analysis of 2 case-control studies conducted in 1993-96 and 2003-06.

Setting: England, UK

Participants: 138 SIDS deaths in 2020 compared to 402 SIDS deaths and 1387 age-equivalent surviving controls, combined from previous studies.

Results: The increased vulnerability of SIDS infants identified in previous studies has become more marked. The infants who died in 2020 were younger (median=66 days [iqr:34-118]vs86 days [iqr:52-148], p=0.003) with an increased prevalence of low birthweight (30.5%vs21.6%, p=0.04), pre-term births (29.6%vs19.3%, p=0.012) and admission to a Neonatal Intensive Care Unit (28.7%vs23.5%, p=0.23). The excess of socio-economically deprived families, male infants and high levels of maternal smoking during pregnancy were still evident. Among recent deaths, fewer infants were put down or found on their side, but a similar proportion were put down (15.6%vs14.6%, p=0.81) and found (40.4%vs35.3%, p=0.37) prone, despite population wide risk-reduction advice over three decades. The proportional increase observed in 2003-6 of half the deaths occurring whilst sleeping next to an adult was maintained in 2020, and for the vast majority (90%) this was in hazardous circumstances (adult had consumed alcohol, smoked, slept on a sofa, or the infant was premature or low birthweight and less than 3 months old). More deaths also occurred when there was a disruption in infant care routine compared to previous observations (52.6%vs20.7%, p<0.001).

Conclusions: A more targeted approach is needed with vulnerable families emphasising the importance of sleeping infants on their back and pro-active planning infant sleep when there are disruptions to the normal routine, in particular to avoid hazardous co-sleeping.

For peer review only - http://bmjopen.bmj.com/site/about/guidelines.xhtml

Article Summary

Strengths and limitations of this study

- Data from the case-control studies were collected prospectively with face-to-face interviews shortly after the death or reference sleep and using the same questions so the data were easier to combine.
- Using age-matched surviving controls in these studies provided us with a baseline from which we can establish associated background characteristics and risk factors.
- Given the welcome reduction in SIDS deaths, national data provides us with sufficient numbers to observe patterns among the more recent deaths.
- Only three quarters of all unexpected deaths from 2020 were fully reviewed and available for analysis.
- The lack of recent control data, apart from what is available from national statistics, is a limiting factor.

For peer review only - http://bmjopen.bmj.com/site/about/guidelines.xhtml

Introduction

Sudden infant death syndrome (SIDS) is defined as the unexpected death of a baby aged under one year, which remains unexplained after investigation including a post-mortem, thorough investigation of the death-scene and circumstances of death and review of the clinical history.(1) In England & Wales, identification of risk factors in the infant sleep environment and subsequent uptake of safer sleep advice by caregivers has led to an almost 90% fall in these deaths over the last 35 years.(2)

With the welcome reduction in SIDS the demographic profile has changed, and these tragedies are largely the preserve of families living in socially deprived conditions.(3) The population-wide risk reduction campaigns have been successful but if the modifiable risks previously identified are still occurring amongst current deaths this would suggest a more targeted approach is needed.

We conducted two prospective case-control observational studies in 1993-96 and 2003-06 in England after the “Back to Sleep” campaign in 1991. The major risks identified included infant sleeping position, hazardous sleep locations and social disruption in terms of changes in infant care routine.(3, 4, 5, 6) The only subsequent observational case-control study was conducted in New Zealand in 2012-15, which showed the risk surrounding hazardous bed-sharing was still present.(7)

The National Child Mortality Database (NCMD) was established in 2019, with the core aim to study and analyse the patterns, causes and associated risk factors of child mortality in England. The programme is commissioned by the Healthcare Quality Improvement Partnership (HQIP) as part of the National Clinical Audit and Patient Outcomes Programme (NCAPOP). HQIP is led by a consortium of the Academy of Medical Royal Colleges, the Royal College of Nursing, and National Voices. NCAPOP is funded by NHS England, the Welsh Government and, with some individual projects, other devolved administrations and crown dependencies.

Notification of deaths to NCMD is required by statute within 48 hours. The database is co-ordinated at the University of Bristol, with locality-based Child Death Review Teams (CDRTs) mandated since 2008 (8) to report child deaths. A recent review of the circumstances and supplementary data on the major risk factors for unexplained infant deaths in England has been published.(9) Given the lengthy time it can take to conduct these investigations a cut-off date for completed cases of June 2022 was chosen to report on 70% of unexplained deaths that occurred in 2020, The cohort used in this paper uses a cut- off date of September 2022 when 75% of deaths that occurred in 2020 had been reviewed.

For peer review only - http://bmjopen.bmj.com/site/about/guidelines.xhtml

This paper presents the data from unexplained infant deaths in England in 2020 reported to NCMD, alongside a combined analysis of our two earlier case control studies to assess the relevance of previously identified risk factors and where increased emphasis might be required in risk reduction messaging if we were to adopt a more targeted approach.

Methods

Unexpected and unexplained infant deaths are derived by exclusion; by failing to demonstrate an adequate cause of death. The categorisation as an unexplained death in England is reached using a protocol that includes a detailed clinical history, review of the scene and circumstances of the death (ideally by a health professional together with a police officer), a standardised post-mortem examination by a paediatric pathologist (sometimes together with a forensic pathologist), additional investigations as appropriate, and a final multi-agency child death review meeting.(9)

The outcome of the child death review meeting is further scrutinised, after anonymisation, by the local Child Death Overview Panel (CDOP), which is responsible for overseeing all investigations into child deaths in their area. There are 58 CDOPs in England.

Data Sources

The NCMD 2020 cohort of unexplained infant deaths

We report on the unexpected deaths of infants for which there was no immediately apparent cause of death (e.g. trauma) that occurred between January 1st 2020 and December 31st 2020 that remained unexplained after investigations and the CDOP review had taken place. It is important to note that not all deaths in 2020 had a completed CDOP review (our cut-off was September 2022) which means numbers presented for 2020 will be underestimated. The NCMD has been collecting data since 2019, but the year 2020 was chosen to allow for the majority of deaths to have undergone review, and for newer data collection forms (introduced in 2019) to become established. The supplementary reporting form: sudden unexpected deaths (10) is required for eligible deaths and contains the majority of questions relating to the infant sleep environment.

The CESDI and SWISS combined dataset

The Confidential Enquiry into Stillbirths and Deaths in Infancy (CESDI, 1993-1996) conducted in 5 English regions (Northern, South West, Trent, Wessex and Yorkshire) and the South West Infant Sleep Scene (SWISS, 2003-2006) study were population-based case-control studies collecting data on all sudden and unexpected deaths in infancy over a set timeframe. Very few deaths were missed in

For peer review only - http://bmjopen.bmj.com/site/about/guidelines.xhtml

the study areas. Both studies used similar methods with corresponding questions and categories for responses allowing for variables to be combined. Both studies used surviving control infants from the same population matched or weighted for age. A period of sleep (the ''reference sleep'') was identified in the control infant's life in the 24 hours before the interview, corresponding to the time of day during which the index baby had died. Approval was sought and gained from each regional research ethics committee and by each constituent local research ethics committee for the two studies. Guidelines for strengthening the reporting of observational studies in epidemiology were followed.(11) Details of these studies have been published elsewhere.(4, 5, 6, 12)

Variables definitions

Infant sleeping position

The sleeping position of the infant was a multi-categorical variable with infants being placed supine (on the back), on their side or prone (front) position. Both the position found and put down were recorded and analysed.

Sleep location

A cot-type bed was defined as a cot, crib, Moses basket, 3-sided baby bed attached to adult bed or a travel cot. A baby carrier was defined as a pram, pushchair, bouncy chair, or sling. An adult bed was either a standard UK single or double sized bed.

Co-sleeping

Co-sleeping was defined as an infant who was found sleeping next to an adult whether this was in an adult bed, on a sofa, or in a chair. We have previously analysed the combined data to look specifically at co-sleeping (13) and confirmed significant interactions with hazardous practices (co-sleeping on a sofa or chair, co-sleeping next to an adult who has consumed more than two units of alcohol and co-sleeping next to an adult who habitually smokes).(12) The different hazards associated with co-sleeping were not mutually exclusive; some infants, for instance, slept on a sofa with an adult who smoked and had consumed alcohol. As with previous papers a hierarchical approach of categorisation of co-sleeping was adopted based on the strength of risk reported from the two studies to ease interpretation of the findings. Thus sofa-sharing, quantified as the highest risk from our previous studies, was categorised regardless of whether the parents consumed alcohol or smoked. Bed-sharing and alcohol consumption of two or more units was categorised regardless of smoking status. The remaining category of hazardous bed-sharing were infants who slept next to someone who smokes, or where the infant was vulnerable (defined as infants who were pre-term or low birthweight and < 90 days old). Thus the final category, representing bed-sharing in the absence

For peer review only - http://bmjopen.bmj.com/site/about/guidelines.xhtml

of known hazards, were those parents who did not co-sleep on a sofa, had not consumed two or more units of alcohol, did not smoke, or sleep next to vulnerable infants.

Change in routine

This is defined as a change in routine that may affect infant care for the last sleep; family on holiday, family visit to friends or relatives, party or celebration, having overnight guest(s) stay, taking long journeys etc.

Statistical Methodology

For ease of interpretation the variables were dichotomised using standard definitions, if available, or previous definitions used in the earlier studies. All denominators were provided when quoting prevalence to indicate where data were missing. Chi-square was calculated using Pearson's method, if any expected cell frequencies were less than 5, Fisher's Exact test was used. Continuous data that were not normally distributed were tested using the Mann-Whitney U test and described with medians and inter-quartile ranges (iqr).

Patient and Public Involvement

Families remain at the heart of our research. Our Family Advisory Group has been instrumental in advising us on how to interpret the findings of this analysis, by supporting the development of tools and resources to support families with following safer sleep advice, as part of our wider work.(14)

Results

Ascertainment

Of the 361 sudden and unexpected infant deaths that occurred in England during 2020, 75% (n=270) had been reviewed by a CDOP by September 2022. Of these, 138 were classified as unexpected and unexplained infant deaths (i.e. SIDS), with 133/138 (96.4%) thought to occur during sleep. Of the 405 SIDS infants and 1387 controls in the two case control studies, data were available on the sleep environment in which the infant was found for 402 SIDS infants (99.3%) and 1387 controls (100%).

Background Characteristics

The background characteristics of the SIDS infants and families that distinguished them from surviving controls in the previous observational studies were just as apparent if not more so

For peer review only - http://bmjopen.bmj.com/site/about/guidelines.xhtml

amongst the more recent deaths in 2020 (Table 1). Male preponderance and births to multiparous mothers remained a feature of SIDS whilst infant vulnerability has become more marked. The median age of SIDS deaths is now nearer two months than three months old. Low birthweight, pre-term birth, multiple births and admission to NICU are recognised significant characteristics of SIDS infants compared to age-matched surviving controls but the prevalence for each of these factors was greater in the more recent SIDS deaths. Birth characteristics from England in 2020 (15) suggest low birthweight (6.5%), pre-term births (7.4%) and multiple births (1.4%) have all slightly increased in the population compared to the previous study control data but the prevalence amongst the SIDS deaths in 2020 for these three characteristics was 4-5 fold higher: 36.2% of the SIDS deaths exhibited at least one of these three characteristics. The prevalence of maternal smoking during pregnancy amongst the SIDS deaths seems to have fallen from 65% to 52.1% but the prevalence of maternal smoking at delivery in England has also reduced in this time period by a third from 15.8% in 2006 to 9.6% in 2020 (16), suggesting a 5-6 fold greater prevalence of maternal smokers among current SIDS deaths. The median maternal age among the SIDS deaths has increased from 24 years in previous studies to 27 years in 2020 but in the same time period the average age for first-time mothers in England & Wales has increased from 27.7 years in 2007(17) to 30.7 years in 2020.(15) Data from 2020 in England & Wales suggest 15.7% of births were to mothers aged <25 years (15) compared to 46/131 (35.1%) among the SIDS mothers in the NCMD cohort suggesting young maternal age is still a factor. Ethnicity was difficult to measure because the status in the observational studies was taken from the parents whilst in the NCMD data it was recorded for the infant, thus robust estimates are only available for those categorised as 'white'. In 2020, this category was prevalent in three quarters of the SIDS deaths, 12.7% of infants were of mixed ethnic origin, 7.5% were 'Black or Black British' and 6% were 'Asian or Asian British'. It was also difficult to compare socio-economic status as different metrics were used. In the NCMD data 41.3% of the SIDS families were in the most deprived quintile as measured by the deprivation index based on postcode. Socio -economic status in the observational studies was measured using occupational classification, 17.4% of the control families were classified as semi-skilled, unskilled or unemployed compared to 44.1% of the SIDS families. Thus marked socio-economic deprivation among SIDS deaths (however measured) seems to have been maintained.

Factors in the Infant Sleeping Environment

Amongst the recent SIDS deaths more infants were placed on their back for the last sleep and fewer on their side compared to previous observations, but the same proportion were placed on their front; the proportion found on their front had increased (Table 2). In the NCMD cohort, significantly fewer of the SIDS infants were found in a cot-type bed and significantly more in an adult bed either

For peer review only - http://bmjopen.bmj.com/site/about/guidelines.xhtml

sharing the sleeping surface or sleeping alone. More than half of the SIDS infants in 2020 were sleeping next to an adult and 63/70 (90%) of these in hazardous conditions; co-sleeping on a sofa or chair, next to an adult who had consumed alcohol or smoked or an adult sleeping next to a young infant (< 3 months old) who was born prematurely (<37 weeks) or with low birthweight (<2500g). Illegal drug consumption (mainly cannabis and opioids) prior to the last sleep was not collected in the CESDI study but was collected in the SWISS study and 13% of the SIDS infants slept next to an adult who had consumed these drugs compared to 3.4% of the controls. In 2020 of those families who answered this question 11/50 (22%) had consumed drugs prior to sleeping with the infant. In previous studies a change in infant care routine for the last sleep was a significant predictor and this was even more marked in recent deaths; occurring in 53% of the SIDS deaths in 2020.

Discussion

Despite three decades of advising parents to place young infants on their back for sleep in England, the proportion put down and found prone remains high amongst SIDS deaths. Of those found prone half were less than 4 months old, an age, in terms of infant physiology, where it is particularly difficult for infants to extricate themselves from this position. More than half the SIDS deaths in 2020 occurred when the infant was sharing a sleep surface with an adult, predominantly in hazardous circumstances. Current advice in the UK acknowledges that bed-sharing supports breastfeeding (18) and can happen both in planned and unplanned ways.(19) Health professionals are encouraged to describe the circumstances which make bed-sharing more risky, namely in the presence of alcohol, smoking or with a vulnerable (low birthweight or premature) infant.(13) They also include advice to avoid bed-sharing where there has been any drug use, or medications that can impair arousability, and our findings suggest this needs to be emphasised. Bed-sharing with infants less than 3 months old who were born prematurely or with low birthweight also needs to be emphasised along with not leaving infants to sleep alone in an adult bed. The finding that very low numbers of deaths occur outside of these known risk factors supports the current continuation of the advice to emphasise when not to bed-share.

We have previously reported major changes in the epidemiology of SIDS in 2006.(20) Between 1984 and 2003 in Avon the proportion of SIDS victims from socio-economically deprived families, vulnerable infants (low birthweight, pre-term, multiple births) and infants exposed to tobacco smoke increased over time. During this 20-year period the national SIDS rate fell almost 10-fold from 2.3 deaths to 0.26 per 1000 livebirths; the number of co-sleeping deaths also halved across the two decades but proportionally increased from 12% of all SIDS deaths to 50%. Our case-control study

For peer review only - http://bmjopen.bmj.com/site/about/guidelines.xhtml

conducted in 2003-6 reported that 54% of the SIDS deaths occurred whilst sleeping next to an adult and the median age of SIDS had fallen to 66 days. Recent findings from the NCMD in 2020 have confirmed these earlier findings regarding bed-sharing and infant age and that social deprivation remains a key feature of overall infant and child mortality.(21) Observations of all unexpected infant deaths in 2020 suggests deprivation is still a strong characteristic and the prevalence of infant vulnerability has risen; over a third of the current deaths were either low birthweight, pre-term or a multiple birth. The prevalence of maternal smoking has fallen from 65% in the previous studies to 52% amongst the recent SIDS deaths but is still extremely high, five-fold higher than the levels of maternal smoking at delivery nationally(16) in England; suggesting this is still a strong predictor of these deaths.

The persistence of SIDS risk factors amongst current deaths despite national risk reduction campaigns is not unique to the UK. In the Netherlands,(22) Spain (23), Germany (24), and Australia (25) the proportion of infants placed in a non-supine position is increasing whilst a survey in Norway (26) suggests hazardous bed-sharing is far more common than initially anticipated. In a case-control study conducted in New Zealand in 2012-2015 hazardous bed-sharing especially the interaction with maternal smoking was cited as the greatest risk for unexpected infant deaths.(27)

Strengths and Limitations

Given the prospective nature of the case-control studies, the detailed data collected shortly after the death or reference sleep, using face to face interviews and using the same questions so the data were easier to combine can be seen as strengths of our investigations. The deaths were also carefully classified using multi-disciplinary panels for both studies and the recent cohort. Only 75% of the deaths in 2020 had been reviewed by the chosen cut-off date (partially due to delays caused by the pandemic) and lack of recent control data can be seen as limitations although routinely collected characteristics surrounding the birth at the national level alleviates some of this problem. Socio-economic deprivation was measured differently in the studies compared to the recent cohort but both measures indicate a significant proportion of SIDS families are in the most deprived grouping. Observing changes in different ethnic groups was limited.

Recommendations

The NCMD is a unique resource that can monitor the known risks in the infant sleep environment on an annual basis and could provide evidence of where prevention efforts should focus. Certainly, the finding that almost half of the deaths occur in hazardous co-sleeping circumstances should be a call to all those involved in improving the uptake of safer sleep advice to find ways to support families to

For peer review only - http://bmjopen.bmj.com/site/about/guidelines.xhtml

reduce the risks for their infants from these situations. Sofa sharing and bed-sharing in the presence of an adult who has consumed alcohol or taken drugs should be the first priorities. The finding that changes to the infant care routine are increasingly prevalent in deaths, and that combinations of risk factors make the situation worse,(28) suggests that prevention efforts may also benefit from interventions that directly focus on planning for safety during times of disruption to the normal routine. Our work with vulnerable families has shown that parents appreciate individual tailored advice that provides reasons, giving the how and why certain situations can increase risks for a baby.(29, 30, 31) The increased vulnerability exhibited amongst current SIDS deaths and continued exposure to several modifiable risk factors within the infant sleep environment despite widespread population-based risk-reduction campaigns suggests targeting resources to families most in need may reduce deaths further. Our specific recommendations based on these findings, are:

1. National SIDS risk reduction campaigns should focus on renewed efforts to emphasise the need for all babies to be put down for sleep on their backs and avoid hazardous co-sleeping; in particular to avoid using sofas or consuming alcohol or drugs before bed-sharing.

2. Families with infants at increased risk should be provided with targeted intensive support, particularly in the first few months of a baby's life, to address the barriers to following safer sleep advice, and plan for infant sleep safety during times of disruption to the normal routine.

3. Annual monitoring by CDOPs and the NCMD should include reporting on background characteristics of families affected by unexpected infant deaths, track sleep environment risk factors present in the deaths each year and provide geographical 'heat maps' showing areas that may need more intensive intervention and support.

Data availability statement

Data are available upon reasonable request. Aggregate data may be available on request to the National Child Mortality Database, and subject to approval by HQIP.

Funding

Dr Anna Pease, Research Fellow, NIHR300820, is funded by the NIHR for this research project. The views expressed in this publication are those of the author(s) and not necessarily those of the NIHR, NHS or the UK Department of Health and Social Care. The National Child Mortality Database (NCMD) Programme is commissioned by the Healthcare Quality Improvement Partnership (HQIP) as part of

For peer review only - http://bmjopen.bmj.com/site/about/guidelines.xhtml

the National Clinical Audit and Patient Outcomes Programme (NCAPOP). HQIP is led by a consortium of the Academy of Medical Royal Colleges, the Royal College of Nursing, and National Voices. Its aim is to promote quality improvement in patient outcomes. HQIP holds the contract to commission, manage and develop the National Clinical Audit and Patient Outcomes Programme (NCAPOP), comprising around 40 projects covering care provided to people with a wide range of medical, surgical and mental health conditions. NCAPOP is funded by NHS England, the Welsh Government and, with some individual projects, other devolved administrations and crown dependencies www.hqip.org.uk/national-programmes. NHS England provided additional funding to the NCMD to enable rapid set up of the real-time surveillance system and staff time to support its function but had no input into the data analysis or interpretation.

Contributor Statement

AP: Accepts full responsibility for the work and/or the conduct of the study, had access to the data, and controlled the decision to publish. NT contributed to analysis and interpretation of data, drafting and review of the manuscript. JI participated in the study concept and design, contributed to interpretation of data, drafting and review of the manuscript. PF participated in the study concept and design, contributed to data acquisition, interpretation of data, drafting and review of the manuscript. KP participated in the study concept and design, contributed to interpretation of data, drafting and review of the manuscript. TW participated in the study concept and design, contributed to data acquisition, interpretation of data, drafting and review of the manuscript. VS participated in the study concept and design, contributed to data acquisition, interpretation of data, drafting and review of the manuscript. KP participated in the study concept and design, contributed to interpretation of data, drafting and review of the manuscript. KL participated in the study concept and design, contributed to data acquisition, interpretation of data, drafting and review of the manuscript. BA participated in the study concept and design, contributed to interpretation of data, drafting and review of the manuscript. PB participated in the study concept and design, contributed to data acquisition, analysis, interpretation of data, drafting and review of the manuscript. All authors have seen and approved the final version of the manuscript.

For peer review only - http://bmjopen.bmj.com/site/about/guidelines.xhtml

References

1. Beckwith JB, editor Discussion of terminology and definition of the sudden infant death syndrome. Proc 2nd Int Congress on Causes of Sudden Death in Infants, 1969; 1969: Washington Univ. Washington Press.
2. Office for National Statistics. Unexplained deaths in infancy, England and Wales: 2020. . ONS Statistical Bulletin; 2022.
3. Fleming PJ, Blair PS, Ward Platt M, Tripp J, Smith IJ, CESDI SUDI Research Group. Sudden infant death syndrome and social deprivation: assessing epidemiological factors after post-matching for deprivation. Paediatric and perinatal epidemiology. 2003;17(3):272-80.
4. Blair PS, Sidebotham P, Evason-Coombe C, Edmonds M, Heckstall-Smith EM, Fleming P. Hazardous cosleeping environments and risk factors amenable to change: case-control study of SIDS in south west England. Bmj. 2009;339.
5. Fleming PJ, Blair PS, Bacon C, Bensley D, Smith I, Taylor E, et al. Environment of infants during sleep and risk of the sudden infant death syndrome: results of 1993-5 case-control study for confidential inquiry into stillbirths and deaths in infancy. Bmj. 1996;313(7051):191-5.
6. Blair PS, Mitchell E, Fleming PJ, Smith IJ, Platt MW, Young J, et al. Babies sleeping with parents: case-control study of factors influencing the risk of the sudden infant death syndrome. Commentary: Cot death—the story so far. Bmj. 1999;319(7223):1457-62.
7. Mitchell EA, Thompson JM, Zuccollo J, MacFarlane M, Taylor B, Elder D, et al. The combination of bedsharing and maternal smoking leads to a greatly increased risk of sudden unexpected death in infancy: the New Zealand Nationwide SUDI case control study. New Zealand Medical Journal. 2017;130(1456):52-64.
8. UK Government. Working Together to Safeguard Children. In: Department for Education, editor. 2010.
9. HM Government. Child death review: statutory and operational guidance (England). HM Government (UK) London; 2018.
10. Department of Health and Social Care. Child death reviews: forms for reporting child deaths. 2014.
11. Malta M, Cardoso LO, Bastos FI, Magnanini MMF, Silva CMFPd. STROBE initiative: guidelines on reporting observational studies. Revista de saude publica. 2010;44:559-65.
12. Blair PS, Sidebotham P, Pease A, Fleming PJ. Bed-sharing in the absence of hazardous circumstances: Is there a risk of sudden infant death syndrome? An analysis from two case-control studies conducted in the UK. PLoS One. 2014;9(9):e107799.
13. National Institute for Health and Care Excellence. Postnatal care: Quality standard [QS37] Published: 16 July 2013 Last updated: 27 September 2022.
14. Pease A, Ali B, Ingram J, Blair PS, Fleming PJ. Baby Sleep Research 2023 [Available from: https://babysleepresearch.co.uk/.
15. Office for National Statistics. Birth characteristics in England and Wales: 2020. 2022.
16. Royal College of Paediatrics and Child Health. State of Child Health: Smoking During Pregnancy. 2020.
17. Office for National Statistics. Birth characteristics in England and Wales: 2017. 2019.
18. Ball HL, Howel D, Bryant A, Best E, Russell C, Ward-Platt M. Bed-sharing by breastfeeding mothers: Who bed-shares and what is the relationship with breastfeeding duration? Acta Paediatrica, International Journal of Paediatrics. 2016;105(6):628-34.
19. The Lullaby Trust. Co-sleeping with your baby 2022 [Available from: https://www.lullabytrust.org.uk/safer-sleep-advice/co-sleeping/.
20. Blair PS, Sidebotham P, Berry PJ, Evans M, Fleming PJ. Major epidemiological changes in sudden infant death syndrome: a 20-year population-based study in the UK. The Lancet. 2006;367(9507):314-9.

For peer review only - http://bmjopen.bmj.com/site/about/guidelines.xhtml

21. Odd D, Stoianova S, Sleap V, Williams T, Cook N, McGeehan L, et al. Child Mortality and Social Deprivation: National Child Mortality Database Programme Thematic Report. National Child Mortality Database,; 2021.
22. Kanits F, L'Hoir MP, Boere-Boonekamp MM, Engelberts AC, Feskens EJ. Renewed attention needed for prevention of Sudden Unexpected Death in Infancy in the Netherlands. Frontiers in pediatrics. 2021:1421.
23. Ruiz-Botia I, Cassanello-Peñarroya P, Díez-Izquierdo A, Martínez-Sánchez JM, Balaguer-Santamaria A. Sudden infant death syndrome: Do the parents follow the recommendations? Anales de Pediatría (English Edition). 2020;92(4):222-8.
24. Gaertner VD, Malfertheiner SF, Postpischil J, Brandstetter S, Seelbach-Göbel B, Apfelbacher C, et al. Implementation of safe infant sleep recommendations during night-time sleep in the first year of life in a German birth cohort. Scientific Reports. 2023;13(1):875.
25. Staton S, Pattinson C, Smith S, Pease A, Blair P, Young J, et al. Observed compliance with safe sleeping guidelines in licensed childcare services. Archives of Disease in Childhood. 2019;104(12):1193-7.
26. Osberg S, Kalstad TG, Stray-Pedersen A. Norwegian parents avoid placing infants in prone sleeping positions but frequently share beds in hazardous ways. Acta Paediatr. 2021;110(7):2119-25.
27. MacFarlane ME, Thompson JM, Wilson J, Lawton B, Taylor B, Elder DE, et al. Infant Sleep Hazards and the Risk of Sudden Unexpected Death in Infancy. The Journal of Pediatrics. 2022.
28. Blair P, Platt MW, Smith IJ, Fleming PJ. Sudden infant death syndrome and sleeping position in pre-term and low birth weight infants: an opportunity for targeted intervention. Archives of Disease in Childhood. 2006;91(2):101-6.
29. Pease A, Blair PS, Ingram J, Fleming P. Conversations with families about reducing the risk of sudden infant death syndrome. Journal of Health Visiting. 2019;7(5):226-31.
30. Pease A, Garstang JJ, Ellis C, Watson D, Ingram J, Cabral C, et al. Decision-making for the infant sleep environment among families with children considered to be at risk of sudden unexpected death in infancy: a systematic review and qualitative metasynthesis. BMJ paediatrics open. 2021;5(1).
31. Pease A, Ingram J, Blair PS, Fleming PJ. Factors influencing maternal decision-making for the infant sleep environment in families at higher risk of SIDS: A qualitative study. BMJ Paediatrics Open. 2017;1.

For peer review only

For peer review only - http://bmjopen.bmj.com/site/about/guidelines.xhtml

Table 1: Comparison of infant and family characteristics (combined study data vs NCMD)

		Data from Observational Studies				Data from NCMD		1) vs 2)[1]
**Variable**	***Category***	**1) SIDS**		**Surviving Controls**		**2) SIDS**		**P-value**
		**n/N**	**%**	**n/N**	**%**	**n/N**	**%**	
**Gender**	*Male*	254/402	63.2%	728/1387	52.5%	86/138	62.3%	0.85
**Birthweight**	*<2500g*	87/402	21.6%	68/1379	4.9%	39/128	30.5%	0.04
**Parity**	*> 1 child*	284/402	71.1%	794/1387	57.2%	87/126	69.1%	0.67
**Gestational age**	*Pre-term (<37 weeks)*	77/400	19.3%	72/1375	5.2%	40/135	29.6%	0.012
**Multiple birth**	*Twin or triplet*	18/402	4.5%	13/1387	0.9%	11/137	8.0%	0.12
**Admission to NICU[2]**	*At birth*	94/400	23.5%	99/1378	7.2%	39/136	28.7%	0.23
**Maternal smoking**	*During Pregnancy*	258/398	64.8%	360/1386	26.0%	62/119	52.1%	0.012
**Ethnicity**	*White[3]*	341/389	87.7%	1164/1378	91.7%	97/130	74.6%	<0.001
		**median**	**iqr**	**median**	**iqr**	**median**	**iqr**	
**Infant age**	*At death/ref sleep*	86 days	52-148	100 days	62-162	66 days	34-118	0.003[4]
**Maternal age**	*At death/ref sleep*	24 years	[21-29]	28 years	24-31	27 years	22-32	<0.001[4]

[1] Comparing prevalence of SIDS from the observational studies with SIDS from NCMD 2020.
[2] Neonatal Intensive Care Unit
[3] Infant ethnicity was not recorded in the observational studies, white ethnicity was derived from ethnicity of mother and biological father but no further breakdown in ethnicity grouping could be made
[4] Mann-Whitney U test

For peer review only

For peer review only - http://bmjopen.bmj.com/site/about/guidelines.xhtml

Table 2: Comparison of risk factors in the sleeping environment (combined study data vs NCMD)

		Data from Observational Studies				Data from NCMD		1) vs 2)[1]
**Variable**	***Category***	**1) SIDS**		**Surviving Controls**		**2) SIDS**		**p-value**
		**n/N**	**%**	**n/N**	**%**	**n/N**	**%**	
**Sleeping Position put down**	*Supine*	191/391	48.8%	968/1382	70.0%	61/90	67.8%	0.001
	Side	143/391	36.6%	370/1382	26.8%	15/90	16.7%	<0.001
	Prone	57/391	14.6%	44/1382	3.2%	14/90	15.6%	0.81
**Sleeping Position found**	*Supine*	159/382	41.6%	1106/1333	83.0%	47/89	52.8%	0.06
	Side	88/382	23.0%	141/1333	10.6%	6/89	6.7%	<0.001
	Prone	135/382	35.3%	86/1333	6.5%	36/89	40.4%	0.37
**Sleeping Location**	*Cot-type bed[2]*	202/395	51.1%	1021/1383	73.8%	42/132	31.8%	<0.001
	Baby Carrier[3]	22/395	5.6%	94/1383	6.8%	4/132	3.0%	0.24
	Car Seat	3/395	0.8%	14/1383	1.0%	1/132	0.8%	1.0[10]
	Adult bed (sharing)	112/395	28.4%	206/1383	14.9%	55/132	41.7%	0.004
	Adult bed (alone)	16/395	4.1%	7/1383	0.5%	12/132	9.1%	0.03
	Sofa/chair (sharing)	33/395	8.4%	7/1383	0.5%	15/132	11.4%	0.30
	Sofa/chair (alone)	6/395	1.5%	28/1383	2.0%	1/132	0.8%	0.69[10]
	Other[4]	1/395	0.3%	6/1383	0.4%	2/132	1.5%	0.16[10]
**Co-sleeping for the last sleep**	*Not co-sleeping*	252/397	63.5%	1174/1386	84.7%	62/132	47.0%	
	Any co-sleeping	145/397	36.5%	212/1386	15.3%	70/132	53.0%	<0.001
	Hazardous co-sleeping[5]:							
	On a sofa/chair	33/397	8.3%	7/1386	0.5%	15/132	11.4%	0.29
	Alcohol consumed	29/397	7.3%	12/1386	0.9%	18[6]/132	13.6%	0.03
	Smoking or vulnerable[7]	65/397	16.4%	66/1386	4.8%	30/132	22.7%	0.10
	Co-sleeping no hazards	18/397	4.5%	127/1386	9.2%	7[8]/132	5.3%	0.72
**Change in routine**	*For the last sleep[9]*	81/391	20.7%	171/1383	12.4%	30/57	52.6%	<0.001

[1] Comparing prevalence of SIDS from the observational study with SIDS from NCMD 2020.
[2] Includes, cot, crib, Moses basket, 3-sided baby bed attached to adult bed or a travel cot.
[3] Includes pram, pushchair, bouncy chair or sling.
[4] Includes on floor, in an electronic settling device, waterbed, pod, nest or bean bag.
[5] Consumption of illegal drugs (cannabis/opioids) prior to co-sleeping was not recorded in the CESDI study. In the SWISS study 10/77 cases (13.0%) slept next to an adult who had taken illegal drugs compared to 3/87 of the controls (3.4%). In the NCMD cohort it was 11/50 (22.0%) of those who were asked this question.
[6] This question was only asked for 50 of the 70 co-sleepers so will be an underestimate.
[7] Adult co-sleeper smoked or infant was vulnerable (infants who were pre-term or low birthweight and < 90 days old).
[8] One infant co-slept with another child, one infant slept next to an adult who had taken cannabis and for the remaining five infants the question on alcohol consumption was not asked.
[9] Change in routine involving the infant (e.g. on holiday, visit to friends or relatives, party, overnight guest(s), long journey etc)
[10] Using Fisher's Exact test

For peer review only

For peer review only - http://bmjopen.bmj.com/site/about/guidelines.xhtml

For peer review only

For peer review only - http://bmjopen.bmj.com/site/about/guidelines.xhtml

STROBE Statement—Checklist of items that should be included in reports of ***case-control studies***

	Item No	Recommendation	Page No
**Title and abstract**	1	(*a*) Indicate the study's design with a commonly used term in the title or the abstract	2
		(*b*) Provide in the abstract an informative and balanced summary of what was done and what was found	2
**Introduction**			
Background/rationale	2	Explain the scientific background and rationale for the investigation being reported	4
Objectives	3	State specific objectives, including any prespecified hypotheses	4/5
**Methods**			
Study design	4	Present key elements of study design early in the paper	5
Setting	5	Describe the setting, locations, and relevant dates, including periods of recruitment, exposure, follow-up, and data collection	5
Participants	6	(*a*) Give the eligibility criteria, and the sources and methods of case ascertainment and control selection. Give the rationale for the choice of cases and controls	5
		(*b*) For matched studies, give matching criteria and the number of controls per case	5
Variables	7	Clearly define all outcomes, exposures, predictors, potential confounders, and effect modifiers. Give diagnostic criteria, if applicable	6/7
Data sources/ measurement	8*	For each variable of interest, give sources of data and details of methods of assessment (measurement). Describe comparability of assessment methods if there is more than one group	6/7
Bias	9	Describe any efforts to address potential sources of bias	NA
Study size	10	Explain how the study size was arrived at	NA
Quantitative variables	11	Explain how quantitative variables were handled in the analyses. If applicable, describe which groupings were chosen and why	7
Statistical methods	12	(*a*) Describe all statistical methods, including those used to control for confounding	7
		(*b*) Describe any methods used to examine subgroups and interactions	NA
		(*c*) Explain how missing data were addressed	NA
		(*d*) If applicable, explain how matching of cases and controls was addressed	NA
		(*e*) Describe any sensitivity analyses	NA
**Results**			
Participants	13*	(a) Report numbers of individuals at each stage of study—eg numbers potentially eligible, examined for eligibility, confirmed eligible, included in the study, completing follow-up, and analysed	7
		(b) Give reasons for non-participation at each stage	NA
		(c) Consider use of a flow diagram	NA
Descriptive data	14*	(a) Give characteristics of study participants (eg demographic, clinical, social) and information on exposures and potential confounders	7
		(b) Indicate number of participants with missing data for each variable of interest	7
Outcome data	15*	Report numbers in each exposure category, or summary measures of	8/TABLE

For peer review only - http://bmjopen.bmj.com/site/about/guidelines.xhtml

exposure	1

For peer review only

For peer review only - http://bmjopen.bmj.com/site/about/guidelines.xhtml

Main results	16	(*a*) Give unadjusted estimates and, if applicable, confounder-adjusted estimates and their precision (eg, 95% confidence interval). Make clear which confounders were adjusted for and why they were included	TABLE 2
		(*b*) Report category boundaries when continuous variables were categorized	NA
		(*c*) If relevant, consider translating estimates of relative risk into absolute risk for a meaningful time period	NA
Other analyses	17	Report other analyses done—eg analyses of subgroups and interactions, and sensitivity analyses	NA
**Discussion**			
Key results	18	Summarise key results with reference to study objectives	9
Limitations	19	Discuss limitations of the study, taking into account sources of potential bias or imprecision. Discuss both direction and magnitude of any potential bias	10
Interpretation	20	Give a cautious overall interpretation of results considering objectives, limitations, multiplicity of analyses, results from similar studies, and other relevant evidence	10
Generalisability	21	Discuss the generalisability (external validity) of the study results	10
**Other information**			
Funding	22	Give the source of funding and the role of the funders for the present study and, if applicable, for the original study on which the present article is based	11

*Give information separately for cases and controls.

Note: An Explanation and Elaboration article discusses each checklist item and gives methodological background and published examples of transparent reporting. The STROBE checklist is best used in conjunction with this article (freely available on the Web sites of PLoS Medicine at http://www.plosmedicine.org/, Annals of Internal Medicine at http://www.annals.org/, and Epidemiology at http://www.epidem.com/). Information on the STROBE Initiative is available at http://www.strobe-statement.org.

For peer review only

For peer review only - http://bmjopen.bmj.com/site/about/guidelines.xhtml

Changes in background characteristics and risk factors amongst SIDS infants in England: cohort comparisons from 1993 to 2020

Journal:	*BMJ Open*
Manuscript ID	bmjopen-2023-076751.R1
Article Type:	Original research
Date Submitted by the Author:	08-Aug-2023
Complete List of Authors:	Pease, Anna; University of Bristol Medical School, Population Health Sciences<br>Turner, Nicholas; University of Bristol Medical School, Population Health Sciences<br>Ingram, Jenny; University of Bristol Medical School, Population Health Sciences<br>Fleming, Peter; University of Bristol Medical School, Population Health Sciences<br>Patrick, Karen; Royal United Hospital Bath NHS Trust<br>Williams, Tom; University of Bristol Medical School, Translational Health Sciences<br>Sleap, Vicky; University of Bristol Medical School, Translational Health Sciences<br>Pitts, Kieren; University of Bristol, Research IT<br>Luyt, Karen; University of Bristol Medical School, Translational Health Sciences<br>Ali, Becky; University of Bristol Medical School, Population Health Sciences<br>Blair, Peter; University of Bristol Medical School, Population Health Sciences
**Primary Subject Heading**:	Epidemiology
Secondary Subject Heading:	Paediatrics, Public health, Communication
Keywords:	Cot death < PAEDIATRICS, EPIDEMIOLOGY, Community child health < PAEDIATRICS, PUBLIC HEALTH

SCHOLARONE™
Manuscripts

I, the Submitting Author has the right to grant and does grant on behalf of all authors of the Work (as defined in the below author licence), an exclusive licence and/or a non-exclusive licence for contributions from authors who are: i) UK Crown employees; ii) where BMJ has agreed a CC-BY licence shall apply, and/or iii) in accordance with the terms applicable for US Federal Government officers or employees acting as part of their official duties; on a worldwide, perpetual, irrevocable, royalty-free basis to BMJ Publishing Group Ltd ("BMJ") its licensees and where the relevant Journal is co-owned by BMJ to the co-owners of the Journal, to publish the Work in this journal and any other BMJ products and to exploit all rights, as set out in our licence.

The Submitting Author accepts and understands that any supply made under these terms is made by BMJ to the Submitting Author unless you are acting as an employee on behalf of your employer or a postgraduate student of an affiliated institution which is paying any applicable article publishing charge ("APC") for Open Access articles. Where the Submitting Author wishes to make the Work available on an Open Access basis (and intends to pay the relevant APC), the terms of reuse of such Open Access shall be governed by a Creative Commons licence – details of these licences and which Creative Commons licence will apply to this Work are set out in our licence referred to above.

Other than as permitted in any relevant BMJ Author's Self Archiving Policies, I confirm this Work has not been accepted for publication elsewhere, is not being considered for publication elsewhere and does not duplicate material already published. I confirm all authors consent to publication of this Work and authorise the granting of this licence.

For peer review only

Changes in background characteristics and risk factors amongst SIDS infants in England: cohort comparisons from 1993 to 2020

Anna S Pease[1], Nicholas Turner[1], Jenny Ingram[1], Peter J Fleming[1], Karen Patrick[2], Tom Williams[3], Vicky Sleap[3], Kieren Pitts[4], Karen Luyt[3], Becky Ali[1], Peter S Blair[1]

Corresponding author: Dr Anna S Pease, Population Health Sciences, Bristol Medical School, University of Bristol, UK. Email: a.pease@bristol.ac.uk

[1] Population Health Sciences, Bristol Medical School, University of Bristol, UK.

[2] Royal United Hospitals, Bath, UK.

[3] Translational Health Sciences, Bristol Medical School, University of Bristol, Bristol, UK

[4] Research IT, University of Bristol, UK

For peer review only

For peer review only - http://bmjopen.bmj.com/site/about/guidelines.xhtml

Abstract

Objectives: Using the National Child Mortality Database (NCMD), this work aims to investigate background characteristics and risk factors in the sleeping environment associated with SIDS (sudden infant death syndrome) and compare the prevalence with previous English SIDS case-control studies.

Design: Cohort of SIDS in 2020 compared to a combined analysis of 2 case-control studies conducted in 1993-96 and 2003-06.

Setting: England, UK

Participants: 138 SIDS deaths in 2020 compared to 402 SIDS deaths and 1387 age-equivalent surviving controls, combined from previous studies.

Results: The increased vulnerability of SIDS infants identified in previous studies has become more marked. The infants who died in 2020 were younger (median=66 days [iqr:34-118]vs86 days [iqr:52-148], p=0.003) with an increased prevalence of low birthweight (30.5%vs21.6%, p=0.04), and pre-term births (29.6%vs19.3%, p=0.012). The excess of socio-economically deprived families, male infants and high levels of maternal smoking during pregnancy were still evident. Among recent deaths, fewer infants were put down or found on their side, however, there was no significant change in the proportion of infants who were put down (15.6%vs14.6%, p=0.81) and found prone (40.4%vs35.3%, p=0.37), despite population wide risk-reduction advice over three decades. The proportional increase observed in 2003-6 of half the deaths occurring whilst sleeping next to an adult was maintained in 2020, and for the vast majority (90%) this was in hazardous circumstances (adult had consumed alcohol, smoked, slept on a sofa, or the infant was premature or low birthweight and less than 3 months old). More deaths also occurred when there was a disruption in infant care routine compared to previous observations (52.6%vs20.7%, p<0.001).

Conclusions: A more targeted approach is needed with vulnerable families emphasising the importance of sleeping infants on their back and pro-active planning infant sleep when there are disruptions to the normal routine, in particular to avoid hazardous co-sleeping.

For peer review only

For peer review only - http://bmjopen.bmj.com/site/about/guidelines.xhtml

Article Summary

Strengths and limitations of this study

- Data from the case-control studies were collected prospectively with face-to-face interviews shortly after the death or reference sleep and using the same questions so the data were easier to combine.
- Using age-matched surviving controls in these studies provided us with a baseline from which we can establish associated background characteristics and risk factors.
- Given the welcome reduction in SIDS deaths, national data provides us with sufficient numbers to observe patterns among the more recent deaths.
- Only three quarters of all unexpected deaths from 2020 were fully reviewed and available for analysis.
- The lack of recent control data, apart from what is available from national statistics, is a limiting factor.

For peer review only

Introduction

Sudden infant death syndrome (SIDS) is defined as the unexpected death of a baby aged under one year, which remains unexplained after investigation including a post-mortem, thorough investigation of the death-scene and circumstances of death and review of the clinical history.(1) In England & Wales, identification of risk factors in the infant sleep environment and subsequent uptake of safer sleep advice by caregivers has led to an almost 90% fall in these deaths over the last 35 years.(2)

With the welcome reduction in SIDS the demographic profile has changed, and these tragedies are largely the preserve of families living in socially deprived conditions.(3) The population-wide risk reduction campaigns have been successful but if the modifiable risks previously identified are still occurring amongst current deaths this would suggest a more targeted approach is needed.

We conducted two prospective case-control observational studies in 1993-96 and 2003-06 in England after the "Back to Sleep" campaign in 1991. The major risks identified included infant sleeping position, hazardous sleep locations and social disruption in terms of changes in infant care routine.(3-6) The only subsequent observational case-control study was conducted in New Zealand in 2012-15, which showed the risk surrounding hazardous bed-sharing was still present.(7)

The National Child Mortality Database (NCMD) was established in 2019, with the core aim to study and analyse the patterns, causes and associated risk factors of child mortality in England. The programme is commissioned by the Healthcare Quality Improvement Partnership (HQIP) as part of the National Clinical Audit and Patient Outcomes Programme (NCAPOP). HQIP is led by a consortium of the Academy of Medical Royal Colleges, the Royal College of Nursing, and National Voices. NCAPOP is funded by NHS England, the Welsh Government and, with some individual projects, other devolved administrations and crown dependencies.

Notification of deaths to NCMD is required by statute within 48 hours. The database is co-ordinated at the University of Bristol, with locality-based Child Death Review Teams (CDRTs) mandated since 2008 (8) to report child deaths. A recent review of the circumstances and supplementary data on the major risk factors for unexplained infant deaths in England has been published.(9) Given the lengthy time it can take to conduct these investigations a cut-off date for completed cases of June 2022 was chosen to report on 70% of unexplained deaths that occurred in 2020, The cohort used in this paper uses a cut- off date of September 2022 when 75% of deaths that occurred in 2020 had been reviewed.

For peer review only - http://bmjopen.bmj.com/site/about/guidelines.xhtml

This paper presents the data from unexplained infant deaths in England in 2020 reported to NCMD, alongside a combined analysis of our two earlier case control studies to assess the relevance of previously identified risk factors and where increased emphasis might be required in risk reduction messaging if we were to adopt a more targeted approach.

Methods

Unexpected and unexplained infant deaths are derived by exclusion; by failing to demonstrate an adequate cause of death. The categorisation as an unexplained death in England is reached using a protocol that includes a detailed clinical history, review of the scene and circumstances of the death (ideally by a health professional together with a police officer), a standardised post-mortem examination by a paediatric pathologist (sometimes together with a forensic pathologist), additional investigations as appropriate, and a final multi-agency child death review meeting.(9)

The outcome of the child death review meeting is further scrutinised, after anonymisation, by the local Child Death Overview Panel (CDOP), which is responsible for overseeing all investigations into child deaths in their area. There are 58 CDOPs in England.

Data Sources

The NCMD 2020 cohort of unexplained infant deaths

We report on the unexpected deaths of infants for which there was no immediately apparent cause of death (e.g. trauma) that occurred between January 1st 2020 and December 31st 2020 that remained unexplained after investigations and the CDOP review had taken place. It is important to note that not all deaths in 2020 had a completed CDOP review (our cut-off was September 2022) which means numbers presented for 2020 will be underestimated. The NCMD has been collecting data since 2019, but the year 2020 was chosen to allow for the majority of deaths to have undergone review, and for newer data collection forms (introduced in 2019) to become established. The supplementary reporting form: sudden unexpected deaths (10) is required for eligible deaths and contains the majority of questions relating to the infant sleep environment.

The CESDI and SWISS combined dataset

The Confidential Enquiry into Stillbirths and Deaths in Infancy (CESDI, 1993-1996) conducted in 5 English regions (Northern, South West, Trent, Wessex and Yorkshire) and the South West Infant Sleep Scene (SWISS, 2003-2006) study were population-based case-control studies collecting data on all sudden and unexpected deaths in infancy over a set timeframe. Very few deaths were missed in

For peer review only - http://bmjopen.bmj.com/site/about/guidelines.xhtml

the study areas. Both studies used similar methods with corresponding questions and categories for responses allowing for variables to be combined. Both studies used surviving control infants from the same population matched or weighted for age. A period of sleep (the ''reference sleep'') was identified in the control infant's life in the 24 hours before the interview, corresponding to the time of day during which the index baby had died. Approval was sought and gained from each regional research ethics committee and by each constituent local research ethics committee for the two studies. Guidelines for strengthening the reporting of observational studies in epidemiology were followed.(11) Details of these studies have been published elsewhere.(4-6, 12)

Exposure variables were similar across all datasets, with only notable differences for ethnicity (which was collected from the parents in the earlier case control studies, and recorded for the infant in the NCMD data), and socio-economic status (which used occupational classification in the earlier case control studies, and Index of Multiple Deprivation score in the NCMD data). Wording of questions relating to the sleep environment were comparable across all three datasets.

Variables definitions

Infant sleeping position

The sleeping position of the infant was a multi-categorical variable with infants being placed supine (on the back), on their side or prone (front) position. Both the position found and put down were recorded and analysed.

Sleep location

A cot-type bed was defined as a cot, crib, Moses basket, 3-sided baby bed attached to adult bed or a travel cot. A baby carrier was defined as a pram, pushchair, bouncy chair, or sling. An adult bed was either a standard UK single or double sized bed.

Co-sleeping

Co-sleeping was defined as an infant who was found sleeping next to an adult whether this was in an adult bed, on a sofa, or in a chair. We have previously analysed the combined data to look specifically at co-sleeping (13) and confirmed significant interactions with hazardous practices (co-sleeping on a sofa or chair, co-sleeping next to an adult who has consumed more than two units of alcohol and co-sleeping next to an adult who habitually smokes).(12) The different hazards associated with co-sleeping were not mutually exclusive; some infants, for instance, slept on a sofa with an adult who smoked and had consumed alcohol. As with previous papers a hierarchical approach of categorisation of co-sleeping was adopted based on the strength of risk reported from

For peer review only - http://bmjopen.bmj.com/site/about/guidelines.xhtml

the two studies to ease interpretation of the findings. Thus sofa-sharing, quantified as the highest risk from our previous studies, was categorised regardless of whether the parents consumed alcohol or smoked. Bed-sharing and alcohol consumption of two or more units was categorised regardless of smoking status. The remaining category of hazardous bed-sharing were infants who slept next to someone who smokes, or where the infant was vulnerable (defined as infants who were pre-term or low birthweight and < 90 days old). Thus the final category, representing bed-sharing in the absence of known hazards, were those parents who did not co-sleep on a sofa, had not consumed two or more units of alcohol, did not smoke, or sleep next to vulnerable infants.

Change in routine

This is defined as a change in routine that may affect infant care for the last sleep; family on holiday, family visit to friends or relatives, party or celebration, having overnight guest(s) stay, taking long journeys etc.

Statistical Methodology

For ease of interpretation the variables were dichotomised using standard definitions, if available, or previous definitions used in the earlier studies. All denominators were provided when quoting prevalence to indicate where data were missing. Chi-square was calculated using Pearson's method, if any expected cell frequencies were less than 5, Fisher's Exact test was used. Continuous data that were not normally distributed were tested using the Mann-Whitney U test and described with medians and inter-quartile ranges (iqr). P values are reported with a significance threshold of .05.

Patient and Public Involvement

Families remain at the heart of our research. Our Family Advisory Group has been instrumental in advising us on how to interpret the findings of this analysis, by supporting the development of tools and resources to support families with following safer sleep advice, as part of our wider work.(14)

Results

Ascertainment

Of the 361 sudden and unexpected infant deaths that occurred in England during 2020, 75% (n=270) had been reviewed by a CDOP by September 2022. Of these, 138 were classified as unexpected and unexplained infant deaths (i.e. SIDS), with 133/138 (96.4%) thought to occur during sleep. Of the 405

For peer review only - http://bmjopen.bmj.com/site/about/guidelines.xhtml

SIDS infants and 1387 controls in the two case control studies, data were available on the sleep environment in which the infant was found for 402 SIDS infants (99.3%) and 1387 controls (100%).

Background Characteristics

The background characteristics of the SIDS infants and families that distinguished them from surviving controls in the previous observational studies were just as apparent if not more so amongst the more recent deaths in 2020 (Table 1). Male preponderance and births to multiparous mothers remained a feature of SIDS whilst infant vulnerability has become more marked. The median age of SIDS deaths is now nearer two months than three months old. Low birthweight, pre-term birth, multiple births and admission to NICU are recognised significant characteristics of SIDS infants compared to age-matched surviving controls but the prevalence for each of these factors was greater in the more recent SIDS deaths, with 36.2% of the SIDS deaths in 2020 exhibiting at least one of these characteristics. The prevalence of maternal smoking during pregnancy amongst the SIDS deaths seems to have fallen from 65% to 52.1% but the prevalence of maternal smoking at delivery in England has also reduced in this time period by a third from 15.8% in 2006 to 9.6% in 2020 (15), suggesting a 5-6 fold greater prevalence of maternal smokers among current SIDS deaths. The median maternal age among the SIDS deaths has increased from 24 years in previous studies to 27 years in 2020 but in the same time period the average age for first-time mothers in England & Wales has increased from 27.7 years in 2007(16) to 30.7 years in 2020.(17) Ethnicity was difficult to measure because the status in the observational studies was taken from the parents whilst in the NCMD data it was recorded for the infant, thus robust estimates are only available for those categorised as ‘white’. In 2020, this category was prevalent in three quarters of the SIDS deaths, 12.7% of infants were of mixed ethnic origin, 7.5% were ‘Black or Black British’ and 6% were ‘Asian or Asian British'. It was also difficult to compare socio-economic status as different metrics were used. In the NCMD data 41.3% of the SIDS families were in the most deprived quintile as measured by the deprivation index based on postcode. Socio -economic status in the observational studies was measured using occupational classification, 17.4% of the control families were classified as semi-skilled, unskilled or unemployed compared to 44.1% of the SIDS families. Thus marked socio-economic deprivation among SIDS deaths (however measured) seems to have been maintained.

Factors in the Infant Sleeping Environment

Amongst the recent SIDS deaths more infants were placed on their back for the last sleep and fewer on their side compared to previous observations, but the same proportion were placed on their

For peer review only - http://bmjopen.bmj.com/site/about/guidelines.xhtml

front; the proportion found on their front had increased (Table 2). In the NCMD cohort, significantly fewer of the SIDS infants were found in a cot-type bed and significantly more in an adult bed either sharing the sleeping surface or sleeping alone. More than half of the SIDS infants in 2020 were sleeping next to an adult and 63/70 (90%) of these in hazardous conditions; co-sleeping on a sofa or chair, next to an adult who had consumed alcohol or smoked or an adult sleeping next to a young infant (< 3 months old) who was born prematurely (<37 weeks) or with low birthweight (<2500g). Illegal drug consumption (mainly cannabis and opioids) prior to the last sleep was not collected in the CESDI study but was collected in the SWISS study and 13% of the SIDS infants slept next to an adult who had consumed these drugs compared to 3.4% of the controls. In 2020 of those families who answered this question 11/50 (22%) had consumed drugs prior to sleeping with the infant. In previous studies a change in infant care routine for the last sleep was a significant predictor and this was even more marked in recent deaths; occurring in 53% of the SIDS deaths in 2020.

Discussion

Despite three decades of advising parents to place young infants on their back for sleep in England, the proportion put down and found prone remains high amongst SIDS deaths. Of those found prone half were less than 4 months old, an age, in terms of infant physiology, where it is particularly difficult for infants to extricate themselves from this position. More than half the SIDS deaths in 2020 occurred when the infant was sharing a sleep surface with an adult, predominantly in hazardous circumstances. Current advice in the UK acknowledges that bed-sharing supports breastfeeding (18) and can happen both in planned and unplanned ways.(19) Health professionals are encouraged to describe the circumstances which make bed-sharing more risky, namely in the presence of alcohol, smoking or with a vulnerable (low birthweight or premature) infant.(13) They also include advice to avoid bed-sharing where there has been any drug use, or medications that can impair arousability, and our findings suggest this needs to be emphasised. Bed-sharing with infants less than 3 months old who were born prematurely or with low birthweight also needs to be emphasised along with not leaving infants to sleep alone in an adult bed. The finding that very low numbers of deaths occur outside of these known risk factors supports the current continuation of the advice to emphasise when not to bed-share.

We have previously reported major changes in the epidemiology of SIDS in 2006.(20) Between 1984 and 2003 in Avon the proportion of SIDS victims from socio-economically deprived families, vulnerable infants (low birthweight, pre-term, multiple births) and infants exposed to tobacco smoke increased over time. During this 20-year period the national SIDS rate fell almost 10-fold from 2.3

For peer review only - http://bmjopen.bmj.com/site/about/guidelines.xhtml

deaths to 0.26 per 1000 livebirths; the number of co-sleeping deaths also halved across the two decades but proportionally increased from 12% of all SIDS deaths to 50%. Our case-control study conducted in 2003-6 reported that 54% of the SIDS deaths occurred whilst sleeping next to an adult and the median age of SIDS had fallen to 66 days. Recent findings from the NCMD in 2020 have confirmed these earlier findings regarding bed-sharing and infant age and that social deprivation remains a key feature of overall infant and child mortality.(21) Observations of all unexpected infant deaths in 2020 suggests deprivation is still a strong characteristic and the prevalence of infant vulnerability has risen; over a third of the current deaths were either low birthweight, pre-term or a multiple birth. Birth characteristics from England in 2020 (17) suggest low birthweight (6.5%), pre-term births (7.4%) and multiple births (1.4%) have all slightly increased in the population compared to the previous study control data but the prevalence amongst the SIDS deaths in 2020 for these three characteristics was 4-5 fold higher. The prevalence of maternal smoking has fallen from 65% in the previous studies to 52% amongst the recent SIDS deaths but is still extremely high, five-fold higher than the levels of maternal smoking at delivery nationally(15) in England; suggesting this is still a strong predictor of these deaths. Data from 2020 in England & Wales also suggest 15.7% of births were to mothers aged <25 years (17) compared to 46/131 (35.1%) among the SIDS mothers in the NCMD cohort suggesting young maternal age is still a factor.

The persistence of SIDS risk factors amongst current deaths despite national risk reduction campaigns is not unique to the UK. In the Netherlands,(22) Spain (23), Germany (24), and Australia (25) the proportion of infants placed in a non-supine position is increasing whilst a survey in Norway (26) suggests hazardous bed-sharing is far more common than initially anticipated. In a case-control study conducted in New Zealand in 2012-2015 hazardous bed-sharing especially the interaction with maternal smoking was cited as the greatest risk for unexpected infant deaths.(27)

Strengths and Limitations

Given the prospective nature of the case-control studies, the detailed data collected shortly after the death or reference sleep, using face to face interviews and using the same questions so the data were easier to combine can be seen as strengths of our investigations. The deaths were also carefully classified using multi-disciplinary panels for both studies and the recent cohort. Only 75% of the deaths in 2020 had been reviewed by the chosen cut-off date (partially due to delays caused by the pandemic) and lack of recent control data can be seen as limitations although routinely collected characteristics surrounding the birth at the national level alleviates some of this problem. Socio-economic deprivation was measured differently in the studies compared to the recent cohort

For peer review only - http://bmjopen.bmj.com/site/about/guidelines.xhtml

but both measures indicate a significant proportion of SIDS families are in the most deprived grouping. Observing changes in different ethnic groups was limited.

Recommendations

The NCMD is a unique resource that can monitor the known risks in the infant sleep environment on an annual basis and could provide evidence of where prevention efforts should focus. Certainly, the finding that almost half of the deaths occur in hazardous co-sleeping circumstances should be a call to all those involved in improving the uptake of safer sleep advice to find ways to support families to reduce the risks for their infants from these situations. Sofa sharing and bed-sharing in the presence of an adult who has consumed alcohol or taken drugs should be the first priorities. The finding that changes to the infant care routine are increasingly prevalent in deaths, and that combinations of risk factors make the situation worse,(28) suggests that prevention efforts may also benefit from interventions that directly focus on planning for safety during times of disruption to the normal routine. Our work with vulnerable families has shown that parents appreciate individual tailored advice that provides reasons, giving the how and why certain situations can increase risks for a baby.(29-31) The increased vulnerability exhibited amongst current SIDS deaths and continued exposure to several modifiable risk factors within the infant sleep environment despite widespread population-based risk-reduction campaigns suggests targeting resources to families most in need may reduce deaths further. Our specific recommendations based on these findings, are:

1. National SIDS risk reduction campaigns should focus on renewed efforts to emphasise the need for all babies to be put down for sleep on their backs and avoid hazardous co-sleeping; in particular to avoid using sofas or consuming alcohol or drugs before bed-sharing.

2. Families with infants at increased risk should be provided with targeted intensive support, particularly in the first few months of a baby's life, to address the barriers to following safer sleep advice, and plan for infant sleep safety during times of disruption to the normal routine.

3. Annual monitoring by CDOPs and the NCMD should include reporting on background characteristics of families affected by unexpected infant deaths, track sleep environment risk factors present in the deaths each year and provide geographical 'heat maps' showing areas that may need more intensive intervention and support.

For peer review only - http://bmjopen.bmj.com/site/about/guidelines.xhtml

Data availability statement

Data are available upon reasonable request. Aggregate data may be available on request to the National Child Mortality Database, and subject to approval by HQIP.

Ethics approval

The NCMD legal basis to collect confidential and personal level data under the Common Law Duty of Confidentiality has been established through the Children Act 2004 Sections M-N, Working Together to Safeguard Children 2018 (https://consult.education.gov.uk/child-protection-safeguarding-and-family-law/working-together-to-safeguard-children-revisions-t/supporting_documents/WorkingTogethertoSafeguardChildren.pdf) and associated Child Death Review Statutory & Operational Guidance https://assets.publishing.service.gov.uk/government/uploads/system/uploads/attachment_data/file/859302/child-death-review-statutory-and-operational-guidance-england.pdf).

Funding

Dr Anna Pease, Research Fellow, NIHR300820, is funded by the NIHR for this research project. The views expressed in this publication are those of the author(s) and not necessarily those of the NIHR, NHS or the UK Department of Health and Social Care. The National Child Mortality Database (NCMD) Programme is commissioned by the Healthcare Quality Improvement Partnership (HQIP) as part of the National Clinical Audit and Patient Outcomes Programme (NCAPOP). HQIP is led by a consortium of the Academy of Medical Royal Colleges, the Royal College of Nursing, and National Voices. Its aim is to promote quality improvement in patient outcomes. HQIP holds the contract to commission, manage and develop the National Clinical Audit and Patient Outcomes Programme (NCAPOP), comprising around 40 projects covering care provided to people with a wide range of medical, surgical and mental health conditions. NCAPOP is funded by NHS England, the Welsh Government and, with some individual projects, other devolved administrations and crown dependencies www.hqip.org.uk/national-programmes. NHS England provided additional funding to the NCMD to enable rapid set up of the real-time surveillance system and staff time to support its function but had no input into the data analysis or interpretation.

Competing interests: No competing interest.

Contributor Statement

AP: Accepts full responsibility for the work and/or the conduct of the study, had access to the data, and controlled the decision to publish. NT contributed to analysis and interpretation of data, drafting and review of the manuscript. JI participated in the study concept and design, contributed to interpretation of data, drafting and review of the manuscript. PF participated in the

For peer review only - http://bmjopen.bmj.com/site/about/guidelines.xhtml

study concept and design, contributed to data acquisition, interpretation of data, drafting and review of the manuscript. KP participated in the study concept and design, contributed to interpretation of data, drafting and review of the manuscript. TW participated in the study concept and design, contributed to data acquisition, interpretation of data, drafting and review of the manuscript. VS participated in the study concept and design, contributed to data acquisition, interpretation of data, drafting and review of the manuscript. KP participated in the study concept and design, contributed to interpretation of data, drafting and review of the manuscript. KL participated in the study concept and design, contributed to data acquisition, interpretation of data, drafting and review of the manuscript. BA participated in the study concept and design, contributed to interpretation of data, drafting and review of the manuscript. PB participated in the study concept and design, contributed to data acquisition, analysis, interpretation of data, drafting and review of the manuscript. All authors have seen and approved the final version of the manuscript.

For peer review only - http://bmjopen.bmj.com/site/about/guidelines.xhtml

References

1. Beckwith JB, editor Discussion of terminology and definition of the sudden infant death syndrome. Proc 2nd Int Congress on Causes of Sudden Death in Infants, 1969; 1969: Washington Univ. Washington Press.
2. Office for National Statistics. Unexplained deaths in infancy, England and Wales: 2020. . ONS Statistical Bulletin; 2022.
3. Fleming PJ, Blair PS, Ward Platt M, Tripp J, Smith IJ, CESDI SUDI Research Group. Sudden infant death syndrome and social deprivation: assessing epidemiological factors after post-matching for deprivation. Paediatric and perinatal epidemiology. 2003;17(3):272-80.
4. Blair PS, Sidebotham P, Evason-Coombe C, Edmonds M, Heckstall-Smith EM, Fleming P. Hazardous cosleeping environments and risk factors amenable to change: case-control study of SIDS in south west England. Bmj. 2009;339.
5. Fleming PJ, Blair PS, Bacon C, Bensley D, Smith I, Taylor E, et al. Environment of infants during sleep and risk of the sudden infant death syndrome: results of 1993-5 case-control study for confidential inquiry into stillbirths and deaths in infancy. Bmj. 1996;313(7051):191-5.
6. Blair PS, Mitchell E, Fleming PJ, Smith IJ, Platt MW, Young J, et al. Babies sleeping with parents: case-control study of factors influencing the risk of the sudden infant death syndrome. Commentary: Cot death—the story so far. Bmj. 1999;319(7223):1457-62.
7. Mitchell EA, Thompson JM, Zuccollo J, MacFarlane M, Taylor B, Elder D, et al. The combination of bedsharing and maternal smoking leads to a greatly increased risk of sudden unexpected death in infancy: the New Zealand Nationwide SUDI case control study. New Zealand Medical Journal. 2017;130(1456):52-64.
8. UK Government. Working Together to Safeguard Children. In: Department for Education, editor. 2010.
9. HM Government. Child death review: statutory and operational guidance (England). HM Government (UK) London; 2018.
10. Department of Health and Social Care. Child death reviews: forms for reporting child deaths. 2014.
11. Malta M, Cardoso LO, Bastos FI, Magnanini MMF, Silva CMFPd. STROBE initiative: guidelines on reporting observational studies. Revista de saude publica. 2010;44:559-65.
12. Blair PS, Sidebotham P, Pease A, Fleming PJ. Bed-sharing in the absence of hazardous circumstances: Is there a risk of sudden infant death syndrome? An analysis from two case-control studies conducted in the UK. PLoS One. 2014;9(9):e107799.
13. National Institute for Health and Care Excellence. Postnatal care: Quality standard [QS37] Published: 16 July 2013 Last updated: 27 September 2022.
14. Pease A, Ali B, Ingram J, Blair PS, Fleming PJ. Baby Sleep Research 2023 [Available from: https://babysleepresearch.co.uk/.
15. Royal College of Paediatrics and Child Health. State of Child Health: Smoking During Pregnancy. 2020.
16. Office for National Statistics. Birth characteristics in England and Wales: 2017. 2019.
17. Office for National Statistics. Birth characteristics in England and Wales: 2020. 2022.
18. Ball HL, Howel D, Bryant A, Best E, Russell C, Ward-Platt M. Bed-sharing by breastfeeding mothers: Who bed-shares and what is the relationship with breastfeeding duration? Acta Paediatrica, International Journal of Paediatrics. 2016;105(6):628-34.
19. The Lullaby Trust. Co-sleeping with your baby 2022 [Available from: https://www.lullabytrust.org.uk/safer-sleep-advice/co-sleeping/.
20. Blair PS, Sidebotham P, Berry PJ, Evans M, Fleming PJ. Major epidemiological changes in sudden infant death syndrome: a 20-year population-based study in the UK. The Lancet. 2006;367(9507):314-9.
21. Odd D, Stoianova S, Sleap V, Williams T, Cook N, McGeehan L, et al. Child Mortality and Social Deprivation: National Child Mortality Database Programme Thematic Report. National Child Mortality Database,; 2021.

For peer review only - http://bmjopen.bmj.com/site/about/guidelines.xhtml

22. Kanits F, L'Hoir MP, Boere-Boonekamp MM, Engelberts AC, Feskens EJ. Renewed attention needed for prevention of Sudden Unexpected Death in Infancy in the Netherlands. Frontiers in pediatrics. 2021:1421.
23. Ruiz-Botia I, Cassanello-Peñarroya P, Díez-Izquierdo A, Martínez-Sánchez JM, Balaguer-Santamaria A. Sudden infant death syndrome: Do the parents follow the recommendations? Anales de Pediatría (English Edition). 2020;92(4):222-8.
24. Gaertner VD, Malfertheiner SF, Postpischil J, Brandstetter S, Seelbach-Göbel B, Apfelbacher C, et al. Implementation of safe infant sleep recommendations during night-time sleep in the first year of life in a German birth cohort. Scientific Reports. 2023;13(1):875.
25. Staton S, Pattinson C, Smith S, Pease A, Blair P, Young J, et al. Observed compliance with safe sleeping guidelines in licensed childcare services. Archives of Disease in Childhood. 2019;104(12):1193-7.
26. Osberg S, Kalstad TG, Stray-Pedersen A. Norwegian parents avoid placing infants in prone sleeping positions but frequently share beds in hazardous ways. Acta Paediatr. 2021;110(7):2119-25.
27. MacFarlane ME, Thompson JM, Wilson J, Lawton B, Taylor B, Elder DE, et al. Infant Sleep Hazards and the Risk of Sudden Unexpected Death in Infancy. The Journal of Pediatrics. 2022.
28. Blair P, Platt MW, Smith IJ, Fleming PJ. Sudden infant death syndrome and sleeping position in pre-term and low birth weight infants: an opportunity for targeted intervention. Archives of Disease in Childhood. 2006;91(2):101-6.
29. Pease A, Blair PS, Ingram J, Fleming P. Conversations with families about reducing the risk of sudden infant death syndrome. Journal of Health Visiting. 2019;7(5):226-31.
30. Pease A, Garstang JJ, Ellis C, Watson D, Ingram J, Cabral C, et al. Decision-making for the infant sleep environment among families with children considered to be at risk of sudden unexpected death in infancy: a systematic review and qualitative metasynthesis. BMJ paediatrics open. 2021;5(1).
31. Pease A, Ingram J, Blair PS, Fleming PJ. Factors influencing maternal decision-making for the infant sleep environment in families at higher risk of SIDS: A qualitative study. BMJ Paediatrics Open. 2017;1.

For peer review only

For peer review only - http://bmjopen.bmj.com/site/about/guidelines.xhtml

Table 1: Comparison of infant and family characteristics (combined study data vs NCMD)

		Data from Observational Studies				Data from NCMD		1) vs 2)[1]
**Variable**	***Category***	**1) SIDS**		**Surviving Controls**		**2) SIDS**		**P-value**
		**n/N**	**%**	**n/N**	**%**	**n/N**	**%**	
**Gender**	*Male*	254/402	63.2%	728/1387	52.5%	86/138	62.3%	0.85
**Birthweight**	*<2500g*	87/402	21.6%	68/1379	4.9%	39/128	30.5%	0.04
**Parity**	*> 1 child*	284/402	71.1%	794/1387	57.2%	87/126	69.1%	0.67
**Gestational age**	*Pre-term (<37 weeks)*	77/400	19.3%	72/1375	5.2%	40/135	29.6%	0.012
**Multiple birth**	*Twin or triplet*	18/402	4.5%	13/1387	0.9%	11/137	8.0%	0.12
**Admission to NICU[2]**	*At birth*	94/400	23.5%	99/1378	7.2%	39/136	28.7%	0.23
**Maternal smoking**	*During Pregnancy*	258/398	64.8%	360/1386	26.0%	62/119	52.1%	0.012
**Ethnicity**	*White[3]*	341/389	87.7%	1164/1378	91.7%	97/130	74.6%	<0.001
		**median**	**iqr**	**median**	**iqr**	**median**	**iqr**	
**Infant age**	*At death/ref sleep*	86 days	52-148	100 days	62-162	66 days	34-118	0.003[4]
**Maternal age**	*At death/ref sleep*	24 years	[21-29]	28 years	24-31	27 years	22-32	<0.001[4]

[1] Comparing prevalence of SIDS from the observational studies with SIDS from NCMD 2020.
[2] Neonatal Intensive Care Unit
[3] Infant ethnicity was not recorded in the observational studies, white ethnicity was derived from ethnicity of mother and biological father but no further breakdown in ethnicity grouping could be made
[4] Mann-Whitney U test

For peer review only - http://bmjopen.bmj.com/site/about/guidelines.xhtml

Table 2: Comparison of risk factors in the sleeping environment (combined study data vs NCMD)

		Data from Observational Studies				Data from NCMD		1) vs 2)[1]
**Variable**	***Category***	**1) SIDS**		**Surviving Controls**		**2) SIDS**		**p-value**
		**n/N**	**%**	**n/N**	**%**	**n/N**	**%**	
**Sleeping Position put down**	*Supine*	191/391	48.8%	968/1382	70.0%	61/90	67.8%	0.001
	Side	143/391	36.6%	370/1382	26.8%	15/90	16.7%	<0.001
	Prone	57/391	14.6%	44/1382	3.2%	14/90	15.6%	0.81
**Sleeping Position found**	*Supine*	159/382	41.6%	1106/1333	83.0%	47/89	52.8%	0.06
	Side	88/382	23.0%	141/1333	10.6%	6/89	6.7%	<0.001
	Prone	135/382	35.3%	86/1333	6.5%	36/89	40.4%	0.37
**Sleeping Location**	*Cot-type bed[2]*	202/395	51.1%	1021/1383	73.8%	42/132	31.8%	<0.001
	Baby Carrier[3]	22/395	5.6%	94/1383	6.8%	4/132	3.0%	0.24
	Car Seat	3/395	0.8%	14/1383	1.0%	1/132	0.8%	1.0[10]
	Adult bed (sharing)	112/395	28.4%	206/1383	14.9%	55/132	41.7%	0.004
	Adult bed (alone)	16/395	4.1%	7/1383	0.5%	12/132	9.1%	0.03
	Sofa/chair (sharing)	33/395	8.4%	7/1383	0.5%	15/132	11.4%	0.30
	Sofa/chair (alone)	6/395	1.5%	28/1383	2.0%	1/132	0.8%	0.69[10]
	Other[4]	1/395	0.3%	6/1383	0.4%	2/132	1.5%	0.16[10]
**Co-sleeping for the last sleep**	*Not co-sleeping*	252/397	63.5%	1174/1386	84.7%	62/132	47.0%	
	Any co-sleeping	145/397	36.5%	212/1386	15.3%	70/132	53.0%	<0.001
	Hazardous co-sleeping[5]:							
	On a sofa/chair	33/397	8.3%	7/1386	0.5%	15/132	11.4%	0.29
	Alcohol consumed	29/397	7.3%	12/1386	0.9%	18[6]/132	13.6%	0.03
	Smoking or vulnerable[7]	65/397	16.4%	66/1386	4.8%	30/132	22.7%	0.10
	Co-sleeping no hazards	18/397	4.5%	127/1386	9.2%	7[8]/132	5.3%	0.72
**Change in routine**	*For the last sleep[9]*	81/391	20.7%	171/1383	12.4%	30/57	52.6%	<0.001

[1] Comparing prevalence of SIDS from the observational study with SIDS from NCMD 2020.
[2] Includes, cot, crib, Moses basket, 3-sided baby bed attached to adult bed or a travel cot.
[3] Includes pram, pushchair, bouncy chair or sling.
[4] Includes on floor, in an electronic settling device, waterbed, pod, nest or bean bag.
[5] Consumption of illegal drugs (cannabis/opioids) prior to co-sleeping was not recorded in the CESDI study. In the SWISS study 10/77 cases (13.0%) slept next to an adult who had taken illegal drugs compared to 3/87 of the controls (3.4%). In the NCMD cohort it was 11/50 (22.0%) of those who were asked this question.
[6] This question was only asked for 50 of the 70 co-sleepers so will be an underestimate.
[7] Adult co-sleeper smoked or infant was vulnerable (infants who were pre-term or low birthweight and < 90 days old).
[8] One infant co-slept with another child, one infant slept next to an adult who had taken cannabis and for the remaining five infants the question on alcohol consumption was not asked.
[9] Change in routine involving the infant (e.g. on holiday, visit to friends or relatives, party, overnight guest(s), long journey etc)
[10] Using Fisher's Exact test

For peer review only

STROBE Statement—Checklist of items that should be included in reports of ***case-control studies***

	Item No	Recommendation	Page No
**Title and abstract**	1	(*a*) Indicate the study's design with a commonly used term in the title or the abstract	2
		(*b*) Provide in the abstract an informative and balanced summary of what was done and what was found	2
**Introduction**			
Background/rationale	2	Explain the scientific background and rationale for the investigation being reported	4
Objectives	3	State specific objectives, including any prespecified hypotheses	4/5
**Methods**			
Study design	4	Present key elements of study design early in the paper	5
Setting	5	Describe the setting, locations, and relevant dates, including periods of recruitment, exposure, follow-up, and data collection	5
Participants	6	(*a*) Give the eligibility criteria, and the sources and methods of case ascertainment and control selection. Give the rationale for the choice of cases and controls	5
		(*b*) For matched studies, give matching criteria and the number of controls per case	5
Variables	7	Clearly define all outcomes, exposures, predictors, potential confounders, and effect modifiers. Give diagnostic criteria, if applicable	6/7
Data sources/ measurement	8*	For each variable of interest, give sources of data and details of methods of assessment (measurement). Describe comparability of assessment methods if there is more than one group	6/7
Bias	9	Describe any efforts to address potential sources of bias	NA
Study size	10	Explain how the study size was arrived at	NA
Quantitative variables	11	Explain how quantitative variables were handled in the analyses. If applicable, describe which groupings were chosen and why	7
Statistical methods	12	(*a*) Describe all statistical methods, including those used to control for confounding	7
		(*b*) Describe any methods used to examine subgroups and interactions	NA
		(*c*) Explain how missing data were addressed	NA
		(*d*) If applicable, explain how matching of cases and controls was addressed	NA
		(*e*) Describe any sensitivity analyses	NA
**Results**			
Participants	13*	(a) Report numbers of individuals at each stage of study—eg numbers potentially eligible, examined for eligibility, confirmed eligible, included in the study, completing follow-up, and analysed	7
		(b) Give reasons for non-participation at each stage	NA
		(c) Consider use of a flow diagram	NA
Descriptive data	14*	(a) Give characteristics of study participants (eg demographic, clinical, social) and information on exposures and potential confounders	7
		(b) Indicate number of participants with missing data for each variable of interest	7
Outcome data	15*	Report numbers in each exposure category, or summary measures of	8/TABLE

For peer review only

exposure	1

For peer review only

For peer review only - http://bmjopen.bmj.com/site/about/guidelines.xhtml

Main results	16	(*a*) Give unadjusted estimates and, if applicable, confounder-adjusted estimates and their precision (eg, 95% confidence interval). Make clear which confounders were adjusted for and why they were included	TABLE 2
		(*b*) Report category boundaries when continuous variables were categorized	NA
		(*c*) If relevant, consider translating estimates of relative risk into absolute risk for a meaningful time period	NA
Other analyses	17	Report other analyses done—eg analyses of subgroups and interactions, and sensitivity analyses	NA
**Discussion**			
Key results	18	Summarise key results with reference to study objectives	9
Limitations	19	Discuss limitations of the study, taking into account sources of potential bias or imprecision. Discuss both direction and magnitude of any potential bias	10
Interpretation	20	Give a cautious overall interpretation of results considering objectives, limitations, multiplicity of analyses, results from similar studies, and other relevant evidence	10
Generalisability	21	Discuss the generalisability (external validity) of the study results	10
**Other information**			
Funding	22	Give the source of funding and the role of the funders for the present study and, if applicable, for the original study on which the present article is based	11

*Give information separately for cases and controls.

Note: An Explanation and Elaboration article discusses each checklist item and gives methodological background and published examples of transparent reporting. The STROBE checklist is best used in conjunction with this article (freely available on the Web sites of PLoS Medicine at http://www.plosmedicine.org/, Annals of Internal Medicine at http://www.annals.org/, and Epidemiology at http://www.epidem.com/). Information on the STROBE Initiative is available at http://www.strobe-statement.org.

For peer review only
